# Age at onset of training in children with hearing and speech disorders and the analysis of related factors in Turkey

**DOI:** 10.1186/s13052-019-0723-x

**Published:** 2019-10-15

**Authors:** Ayse Sanem Sahli

**Affiliations:** Vocational School of Health Services, Hearing and Speech Training Center, Hacettepe University, 06100, Sıhhiye, Ankara, Turkey

**Keywords:** Children, Hearing, Speech, Hearing loss, Speech disorder, Training, Early diagnosis, Early intervention

## Abstract

**Background:**

Early diagnosis and intervention play a vital role in hearing and speech disorders and the effect of intervention varies according to the age at onset of training of children with such disorders. Aim of this study is to investigate the age at onset of training in children admitted to our center with complaints of hearing and speech disorder, and the related factors.

**Methods:**

In the first phase of the study, data of 473 children admitted to our center between January 2015 and October 2018 with complaints of hearing and speech disorders and no additional disability were retrospectively analyzed. Then, their chronological age, gender, cause of admission, age at onset of training and the effect of factors that may have an impact on the age at onset of training were analyzed statistically. Study data were obtained from patient records.

**Results:**

Of 473 children (350 males and 123 females) admitted to our training center with the complaints of hearing and speech disorders, 252 (53.3%) were presented with speech sound disorders, 90 (19.0%) with stuttering, 87 (18.4%) with delayed speech, 32 (6.8%) with hearing loss and 12 (2.5%) with other causes. Although there was a statistically significant difference between the age at onset of training and the factors; such as cause of admission, parental education level, employment status of the mother, occupation of the father, and socioeconomic status of the family *(p < 0.05),* no statistically significant difference was found between the age at onset of training and gender *(p > 0.05).*

**Conclusions:**

The study revealed that children with hearing loss have the chance of early diagnosis thanks to neonatal hearing screening programs and that they commence their training until the age of 2, which is considered to be a critical period for language and speech development. However, it is an undeniable fact that we have not yet reached the ideal age for the commencement of training (6th month). Similarly, the age of diagnosis and initiation of training is delayed in children with speech disorders due to families’ delayed referral to the training centers.

## Background

Data obtained from the World Health Organization (WHO) show that around 7.5 million children live with hearing loss in the world [1]. Hearing loss is one of the most common congenital health problems in children and affects approximately 3 in every 1000 newborns in the world [2]. A survey conducted by the Turkish Statistical Institute (TSI) in 2010 showed that 5.9% of people with disabilities in Turkey suffer from hearing loss and 9.6% of them are 0–6 years of age and 17.4% are between 7 and 14 [3]. Approximately 1,300,000 babies are born every year in Turkey and 1300–2600 of these infants are diagnosed with congenital hearing loss. Nowadays, it is possible to diagnose hearing loss with Neonatal Hearing Screening Programs. The national neonatal hearing screening programs in Turkey were first launched in 2004. Today, each baby born in Turkey is subjected to the hearing screening program. Babies who fail in screening tests are referred to tertiary referral clinics for advanced audiological evaluation, definitive diagnosis and instrumentation (amplification), and then to relevant institutions and centers for hearing-speech and language education [4–6]. With the onset of newborn hearing screening programs, children with hearing loss have the chance of early diagnosis and intervention (early hearing aid and cochlear implantation, early speech-language training) [7–12]. Early diagnosis enables early intervention and sets the stage for better speech and language development [13–15]. The critical age for early diagnosis and intervention is up to 6 months. Diagnosis of babies born with congenital hearing loss before 6 months of age and initiation of their training, will increase their communication and academic skills, speech and language in particular; enable them to have better language skills by the age of three compared to children with other hearing loss; and to catch up with the hearing abilities as their peers in the following periods [16, 17]. Some researchers reported that children with hearing loss, who were intervened until 3 months of age achieved higher scores in terms of understandable word count and word production than those who did not [18]. In order for early intervention programs to be carried out effectively, children should be brought to the follow-up examinations by the family on a regular basis. A study reported that in 2005, 64% of the children with hearing loss and 46.1% in 2007 had been canceled from regular follow-ups [19]. Another study showed that approximately 20% of families stopped following the intervention program, which can often be attributed to lack of education or instructions, limitations due to the working conditions of parents, financial problems, transportation difficulties, etc. [1, 20].

In Turkey, individuals with speech and language disorders constitute 0.2% of people with disabilities. 25.1% of these individuals are between 0 and 6 years of age, while 37.1% are between 7 and 14 [3]. Due to the widespread of the “wait and see” approach towards speech disorders by many families and clinicians in Turkey, chances of early diagnosis and intervention are often missed which has a negative impact on educational success. Similarly, certain misconceptions and beliefs (*“His/her brother also had delayed speech. Let’s wait, he’ll talk anyhow”, “His/her father was also stuttering, then resolved spontaneously”, “It’s not really necessary to start talking, we understand what he/she means”*) also mislead parents and delay the age at onset of training [21].

Although there are many studies showing that significant progress has been made regarding the early diagnosis of children with hearing loss in the last fifteen years, there is no current and comprehensive study on the age at onset of training in children with hearing and speech disorders. The aim of this study is to investigate the age at onset of training in children admitted to our center with complaints of hearing and speech disorders, and the factors affecting them.

## Method

### Data collection

Firstly, in the study, children and their families who applied to our training center between January 2015 and October 2018 with complaints of hearing and / or speech disorder were identified by means of reviewing the patient records, which was followed by the retrospective analysis of the data of 473 children and their families. Study data were obtained from patient records. Written consents of the parents (their mothers or fathers) were obtained prior to the study. Our center has been operating in the field of diagnosis and rehabilitation of hearing and speech disorders in cooperation with a university since 1992, and is a reference center admitting patients from different regions and cities of Turkey. Of 473 children admitted to our training center with complaints of hearing and speech disorders, 350 (76%) were males and 123 (24%) were females. Children with any additional illnesses and / or disabilities, except for hearing and speech disorder, were excluded from the study. As the first step, the data planned to be obtained from patient records were identified. These data included the chronological age (month), gender, cause of admission to our center, age at admission (month), province of residence, age of the parents, parental education level, employment status of the mother, occupation of the father, and socioeconomic status of the family. The data obtained after scanning the patient records retrospectively were transferred to a computer, which was followed by the statistical analysis of children’s age at onset of training and their mean age according to the cause of admission; correlation between the age of admission and gender; and fluctuation in the age of admission according to the demographic features of the family.

### Data analyses

Statistical analyses were run by using SPSS 22 and statistical significance was defined as *p < 0.05.* Normality of the variables was analyzed through Shapiro–Wilks tests. Descriptive statistics were presented as mean ± standard deviation for continuous variables together with frequency with proportions for categorical variables. Student’s t test was used to compute the differences between two groups. Besides, one-way Anova was also utilized to compute the differences among more than two groups which was followed by a Bonferroni corrected post-hoc test. Relationship between two categorical data was evaluated with either Pearson’s chi-square test or continuity corrected chi-square test. The relationship was assessed with Pearson’s correlation coefficient for continuous variables and Spearman’s correlation coefficient for ordinal variables.

## Results

Of 473 children admitted to our training center with complaints of hearing and speech disorders, 350 (76%) were males and 123 (24%) were females. A total of 252 (53.3%) were presented with speech sound disorders, 90 (19.0%) with stuttering, 87 (18.4%) with delayed speech, 32 (6.8%) with hearing loss and 12 (2.5%) with other causes (central auditory processing disorder, aphasia, apraxia, etc.). The mean chronological age of the children was 92.6 months (Min - Max: 12–188 months, SD: 24.32). Distribution of children according to age and cause of admission is shown in Table 1. The mean age of admission was 48.9 months (Min - Max: 6–150 months, SD: 19.43). Although the age of admission was lower in males (46.3 months) than it was in females (52.4 months), the difference was not statistically significant *(p > 0.05).*

**Table 1:** Distribution of children according to age and cause of admission *(N:473)*

The cause of admission	Age at admission (month)				
	N	Min	Max.	Mean	SD
Speech sound disorders	252	34	116	62.3	27.12
Stuttering	90	31	150	74.5	38.76
Delayed speech	87	20	112	42.4	31.57
Hearing loss	32	6	56	14.8	16.24
Other causes	12	38	148	50.6	22.31

In the study, the mean age at onset of training was 74.5 months for patients with stuttering, 62.3 months for speech disorder, 42.4 months for delayed speech, 14.8 months for hearing loss and 50.6 for other hearing and speech disorders (Table 1). There was a statistically significant difference between the cause and age of admission *(p < 0.01).* The earliest admission was observed in children with hearing loss, whereas the latest admission was found to be in children with stuttering.

Evaluation of the distribution according to the cause of admission and gender showed that the number of males presented with the complaint of stuttering was significantly higher than females, whereas the number of females presented with complaints of hearing loss was statistically and significantly higher than that of males (Table 2) (*p < 0.05).* Although the number of males presented with delayed speech was higher than females and the number of females presented with speech disorder was higher than males, the result was not statistically significant *(p > 0.05).*

**Table 2:** Distribution of children according to gender and the cause of admission *(N:473)*

The cause of admission	Female (N:123)		Male (350)		Total (N:473)		*p*
	*N*	%	*N*	%	*N*	%	
Speech sound disorders	69	56,1	183	52,3	252	53,3	*p* > 0.05
Stuttering	19	15,4	71	20,3	90	19,0	*p* < 0.05
Delayed speech	21	17,1	66	18,9	87	18,4	*p* > 0.05
Hearing loss	13	10,6	19	5,4	32	6,8	*p* < 0.05
Other causes	1	0,8	11	3,1	12	2,5	*p* > 0.05

Table 3 shows the distribution according to parental educational level, employment status of the mother and occupation of the father. Of all the mothers involved in the study, 36.6% (N: 173) were university graduates, while 25.4% (N: 120) were high school, 21.6% (N: 102) were primary school, 11% (N: 52) and were secondary school graduates, and 5.5% (N: 26) had a higher educational level (master’s or doctoral degree). While 60.7% (N: 287) of these mothers were housewives, 39.3% (N: 186) of them were employed. Analysis of the fathers involved in the study revealed that, 39.1% (N: 185) were university graduates, while 28.1% (N: 133) were high school, 13.7% (N: 65) were primary school, and 10.8% (N: 51) were secondary school graduates, and 8.2% (N: 39) had a higher educational level. The majority of fathers were self-employed (49.3%) and civil servants (37%) (Table 3). In the study, socioeconomic statuses of the families were classified as low, moderate and high, based on the level of income in Turkey.

**Table 3:** Distribution according to parental educational level, employment status of the mother and occupation of the father

Educational level	Mother		Father	
	*N*	%	*N*	%
Primary school	102	21,6	65	13,7
Secondary school	52	11,0	51	10,8
High school	120	25,4	133	28,1
University	173	36,6	185	39,1
Master’s or doctoral	26	5,5	39	8,2
Maternal employment status	N	%	–	–
Housewife	287	60,7	**–**	**–**
Employed	186	39,3	**–**	**–**
Paternal occupation	*N*	%	*N*	%
Self-employed	–	–	233	49,3
Civil servant	–	–	175	37,0
Working	–	–	56	11,8
Retired	–	–	9	1,9

In line with this classification, 56.4% (N: 267) of the families were found to have moderate incomes, while 23.7% (N: 112) had low and 19.9% (N: 94) had high incomes.

In the study, the effects of certain factors; such as parental education level, employment status of the mother, occupation of the father, and socioeconomic status of the family, on the age of admission of the children were also evaluated and the following results were reached:

-There was a statistically significant difference between the parental education level and age of admission *(p < 0.001).* It was observed that the increase in parental education level decreases the age of admission.

-There was a statistically significant difference between the employment status of the mother and age of admission *(p: 0.001).* The age at onset of training in the children with working mothers was lower than that of housewives.

-There was a statistically significant difference between the occupation of the father and age of admission *(p: 0.000).* The age at onset of training in the children with retired fathers was higher than that of others.

-There was a statistically significant difference between the socioeconomic status of the family and age of admission *(p < 0.05).* The age at onset of training in the children of families with low socioeconomic status was higher than that of others.

## Discussion

In Turkey, an average of 3 million babies are born each year and nearly 0.1% of these infants continue their lives with hearing loss. This means that nearly 1300 new patients with hearing loss are referred to audiology clinics and intervention centers every year [5, 22].

In a study conducted on a limited number (*N* = 32) of infants with hearing loss in Turkey, it was stated that 14 of the babies were instrumented in the first 3 months, 7 in 6 months, 11 in 12 months and the rest in 6–12 months which was followed by the commencement of training. It was also stated that 65% of the infants were intervened during the targeted period of neonatal hearing screening programs. Despite the promising results obtained in the study, it likely to reflect the overall profile of Turkey, as it was carried out on a limited number of infants in a local area [23]. In our study, the mean age at onset of training in children with hearing loss was found to be 14.8 months. In Turkey, there are limited number of studies conducted on the age of diagnosis, instrumentation and onset of training of children with hearing loss.

In a comprehensive and long-term study carried out on 4521 children with hearing loss, it was reported that parents do not notice the hearing loss in their children until 3.4 years of age and that the age of diagnosis used to be 4.7 years in the 1970s in Turkey. These figures decreased to 1.7 years and 2.8 years, respectively, in the 1990s [24]. Another study conducted in 2005 found that the age when the hearing loss was noticed was 12.5 months, and that it was 19.4 months for diagnosis, 26.5 months for instrumentation, and 33.0 months for the onset of training [25]. In our study, the mean age at onset of training in children with hearing loss was 14.8 months. In a similar study conducted in India in 2014, the mean age of diagnosis was 24.36 months in children with hearing loss and the age at onset of training was 28.36 months [26].

One of the objectives of the neonatal hearing screening programs is to minimize the negative effects of hearing loss on language and speech skills by means of starting training at an early age (no later than 6 months). Therefore, even with the execution of the hearing screening, the achievement of a normal language-speech development is rather difficult, unless early instrumentation and early education are achieved at an ideal age (no later than 6 months) [27]. The Joint Committee on Infant Hearing (JCIH) recommends universal hearing screening by the first month, diagnosis of hearing loss by 3 months, and enrollment in early intervention by 6 months of age. These recommendations are commonly referred to as the Early Hearing Detection and Intervention (EHDI) 1–3-6 guidelines [13, 14]. When the results of this study and the early ones which were conducted on the age of diagnosis and onset of training in children with hearing loss in Turkey are compared, it is found that the age of diagnosing hearing loss in Turkey decreased below 3 months of age with the help of Newborn Hearing Screening Programs. However, the ideal age highlighted by Joint Committee on Infant Hearing to commence the training (earlier than 6 months) has not been achieved yet, even though the age at onset of training has decreased in comparison to early years [23–25].

This is also the case for speech disorders. In our study, the mean age at onset of training in children with speech disorders was 74.5 months for those suffering from stuttering; 62.3 months in patients with speech disorder; and 42.4 months with delayed speech. In Turkey, the intervention in common speech disorders among children is delayed, especially due to false beliefs, approaches and practices, which results in missing the most critical timing for speech-language intervention [21].

In Turkey, although the chances of early diagnosis and intervention in children born with hearing loss is higher thanks to screening programs, parental factors are still the most important determinants regarding the timing of application. This is also the case for speech disorders, since there is no screening program available. Relevant studies state that the most important reason for delaying the early diagnosis and treatment of children are parental disagreement with the appointments. In particular, certain factors such as lack of understanding the concept of early diagnosis and intervention process, lack of instruction, misguidance, and the opinion that the process is unnecessary are the underlying causes of such approaches [1, 20, 23, 26, 28]. In addition, there are studies highlighting the effects of parental education level and socioeconomic status on early intervention. Especially, families with low levels of education and socioeconomic status are also the reasons for the delay in early diagnosis and early intervention [26, 29, 30]. In our study, it was observed that the age at onset of training of children with parents from lower educational background and socioeconomic status was statistically and significantly lower than the others. Similarly, the age at onset of training is lower in children of working mothers, whereas it is higher in children with retired fathers. Although employment status of the mother is a factor that positively affects the socioeconomic level, retirement of the father causes the exact opposite.

## Conclusions

The study showed that children with hearing loss have the chance of early diagnosis thanks to the neonatal hearing screening programs. Besides, these children start their training at 0–2 years of age, which is considered to be a critical period for language and speech development. However, we have not reached the ideal age for the commencement of training (6th month) yet. Similarly, in children with speech disorders, the age of diagnosis and initiation of training is delayed due to families’ delayed referral to the training center. Parental status is the most important factor in terms of conducting early intervention services for children with hearing and speech disorders. Therefore, it is of utmost importance to inform families, especially healthcare professionals in this area, about the importance of early diagnosis and intervention in hearing and speech disorders; so that we can increase the number of studies and help their circulation across the country in order to raise awareness on the matter. In addition, we believe that the development of early childhood language and speech screening programs that are similar to neonatal hearing and screening programs, and their implementation throughout the country, may lead to a significant progress in early diagnosis and intervention of speech disorders.

## Data Availability

Not applicable.

## References

[1] Ravi R, Gunjawate DR, Yerraguntla K (2016). Follow-up in newborn hearing screening - a systematic review. Int J Pediatr Otorhinolaryngol.

[2] Dedhia K, Graham E, Park A (2018). Hearing loss and failed newborn hearing screen. Clin Perinatol.

[3] Turkish Statistical Institute (TSI) (2010). Republic of Turkey Ministry of Family and Social Policies Survey on Problems and Expectations of Disabled People.

[4] Ozbas S (2012). Ulusal Yenidoğan İşitme Taraması Uygulamaları ve Son Gelişmeler. II.

[5] Kemaloğlu YK, Gökdoğan Ç, Gündüz B (2016). Newborn hearing screening outcomes during the first decade of the program in a reference hospital from Turkey. Eur Arch Otorhinolaryngol.

[6] Sağlık Bakanlığı, Ana Çocuk Sağlığı ve Aile Planlaması Genel Müdürlüğü, Yenidoğan tarama programları. VI. Ulusal Ana Çocuk Sağlığı Kongresi, 2011.

[7] Controlled trial of universal neonatal screening for early identification of permanent childhood hearing impairment. Wessex universal neonatal hearing screening trial group. Lancet 1998; 352:1957–1964. 9872244

[8] Russ SA, Rickards F, Poulakis Z (2002). Six year effectiveness of a population based two tier infant hearing screening programme. Arch Dis Child.

[9] Hutt N, Rhodes C (2008). Post-natal hearing loss in universal neonatal hearing screening communities: current limitations and future directions. J Paediatr Child Health.

[10] Yoshinaga-Itano C (2004). Levels of evidence: universal newborn hearing screening (UNHS) and early hearing detection and intervention systems (EHDI). J Commun Disord.

[11] Mehl AL, Thomson V (1998). Newborn hearing screening: the great omission. Pediatrics.

[12] Spivak L, Sokol H, Auerbach C (2009). Newborn hearing screening follow-up: factors affecting hearing aid fitting by 6 months of age. Am J Audiol.

[13] Muse C, Harrison J, Yoshinaga-Itano C, Grimes A, Brookhouser PE, Epstein S (2013). Supplement to the JCIH 2007 position statement: principles and guidelines for early intervention after confirmation that a child is deaf or hard of hearing. Pediatrics.

[14] American Academy of Pediatrics (2007). Joint committee on infant hearing, year 2007 position statement: principles and guidelines for early hearing detection and intervention programs. Pediatrics.

[15] Keren R, Helfand M, Homer C (2002). Projected cost-effectiveness of statewide universal newborn hearing screening. Pediatrics.

[16] Vohr B, Jodoin-Krauzyk J, Tucker R (2008). Early language outcomes of early-identified infants with permanent hearing loss at 12 to 16 months of age. Pediatrics.

[17] Kennedy CR, McCann DC, Campbell MJ (2006). Language ability after early detection of permanent childhood hearing impairment. N Engl J Med.

[18] Yoshinaga-Itano C, Sedey AL, Coulter DK (1998). Language of early- and later-identified children with hearing loss. Pediatrics.

[19] Centers for Disease Control and Prevention (CDC) (2010). Identifying infants with hearing loss - United States, 1999–2007. Morb Mortal Wkly Rep.

[20] Hardonk S, Desnerck G, Loots G, Matthijs L, Hove GV, Kerschaver EV (2011). From screening to care: qualitative analysis of the parental experiences related to screening and the (re) habilitation care for children with congenital deafness in Flanders, Belgium. Volta Rev.

[21] Zengin-Akkuş P, Çelen-Yoldaş T, Kurtipek G, Özmert EN (2018). Speech delay in toddlers: Are they only “late talkers”?. Turk J Pediatr.

[22] Turkish Statistical Institute (TSI), Turkish statistical institute web, 2016. http://www.tuik.gov.tr/PreHaberBultenleri.do?id=27588. Accessed 24 Apr 2019.

[23] Turan Z (2018). Newborn hearing screening programs and their effects on age of diagnosis, hearing aid fitting and education. Abant İzzet Baysal Üniversitesi Eğitim Fakültesi Dergisi.

[24] Belgin E, Akdas F, Boke B, Caglar A (1991). The children population with sensorineural hearing loss in Turkey. Proceedings of the second International Meeting in Audiology for the Mediterranean Countries.

[25] Ozcebe E, Sevinc S, Belgin E (2005). The ages of suspicion, identification, amplification and intervention in children with hearing loss. Int J Pediatr Otorhinolaryngol.

[26] Ansari MS (2014). Assessing parental role as resource persons in achieving goals of early detection and intervention for children with hearing impairment. DCID.

[27] Moeller MP, Carr G, Seaver L, Stredler-Brown A, Holzinger D (2013). Best practices in family-centered early intervention for children who are deaf or hard of hearing: an international consensus statement. J Deaf Stud Deaf Educ.

[28] Hoffman J, Munoz KF, Bradham TS, Nelson L (2011). Loss to follow-up: issues and recommendations. Volta Rev.

[29] Bush M, Hardin B, Rayle C, Lester C, Studts C, Shinn J (2015). Rural barriers to early diagnosis and treatment of infant hearing loss in Appalachia. Otol Neurotol.

[30] Elpers J, Lester C, Shinn JB, Bush ML (2016). Rural family perspectives and experiences with early infant hearing detection and intervention: a qualitative study. J Community Health.

